# Supplementary material for: The interactome of intact mitochondria by cross-linking mass spectrometry provides evidence for coexisting respiratory supercomplexes
Source: Mol Cell Proteomics. 2017 Dec 8;17(2):216–32. doi: 10.1074/mcp.RA117.000470 (PMC5795388; doi:10.1074/mcp.RA117.000470)
Supplement: Supplemental Data [file supp_RA117.000470_133922_0_supp_23978_fzffwf.zip › spectra_annotation/mito_spectra_annotation/56-1-86-1-86-1.pdf]

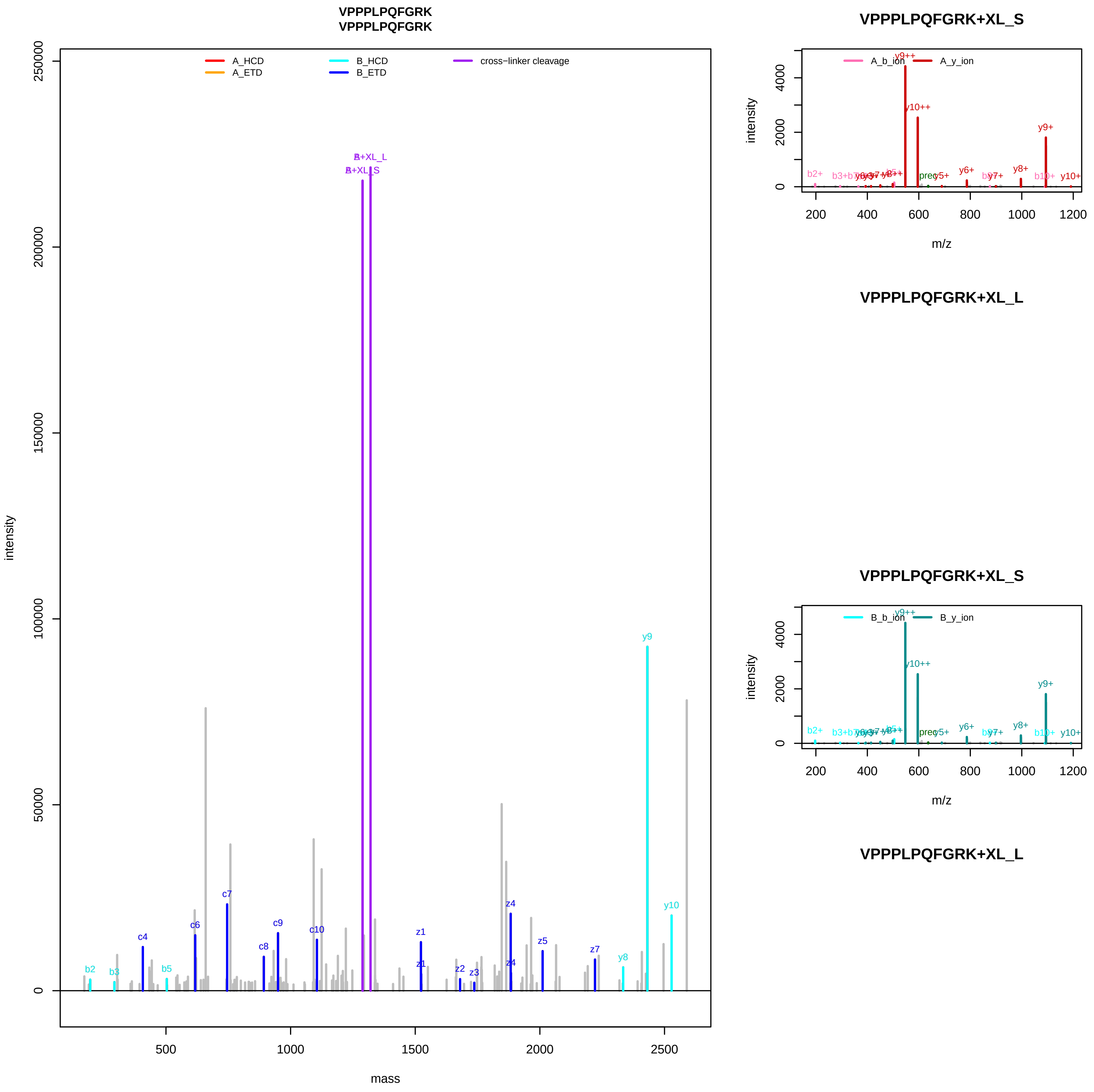
VPPPLPQFGRK
VPPPLPQFGRK
A_HCD
A_ETD
B_HCD
B_ETD
cross−linker cleavage
intensity
mass
B+XL_L
B+XL_S
b2
b3
c4
b5
c6
c7
c8
c9
c10
z1
z2
z3
z4
z5
z7
y8
y9
y10
VPPPLPQFGRK+XL_S
A_b_ion
A_y_ion
B_b_ion
B_y_ion
y9++
y10++
y9+
y8+
y6+
y5+
y10+
b10+
b2+
prec
m/z
VPPPLPQFGRK+XL_L
VPPPLPQFGRK+XL_S
VPPPLPQFGRK+XL_L
